# Visuospatial and Executive Dysfunction in Patients With Acute Kidney Injury, Chronic Kidney Disease, and Kidney Failure: A Multilevel Modeling Analysis

**DOI:** 10.1177/20543581221103100

**Published:** 2022-06-14

**Authors:** Natasha A. Jawa, Jessica A. Vanderlinden, Stephen H. Scott, Jill A. Jacobson, Samuel A. Silver, Rachel Holden, J. Gordon Boyd

**Affiliations:** 1 Centre for Neuroscience Studies, Faculty of Health Sciences, Queen’s University, Kingston, ON, Canada; 2 Department of Biomedical and Molecular Sciences, Queen’s University, Kingston, ON, Canada; 3 Department of Psychology, Queen’s University, Kingston, ON, Canada; 4 Division of Nephrology, Department of Medicine, Queen’s University, Kingston, ON, Canada; 5 Division of Neurology, Department of Medicine, Queen’s University, Kingston, ON, Canada; 6 Department of Critical Care Medicine, Queen’s University, Kingston, ON, Canada; 7 Departments of Medicine (Neurology) and Critical Care, Kingston General Hospital, ON, Canada

**Keywords:** acute kidney injury, chronic kidney disease, cognitive dysfunction, multilevel analysis, neurocognitive test

## Abstract

**Background:**

Neurocognitive impairment is a common finding across the spectrum of kidney disease and carries important consequences for quality of life. We previously demonstrated that robotic technology can identify neurocognitive impairments not readily detectable by traditional testing in patients with acute kidney injury (AKI) and chronic kidney disease (CKD).

**Objective:**

The present study aimed to assess whether these quantifiable deficits in neurocognition differ based on a diagnosis of AKI, CKD, or kidney failure.

**Design:**

This was a cross-sectional analysis of participants previously enrolled in an observational study.

**Setting:**

Patients were enrolled at a tertiary academic hospital, Kingston Health Sciences Centre, Kingston, ON, Canada.

**Patients:**

Adults with AKI, CKD, or kidney failure.

**Measurements:**

Each participant underwent robotic neurocognitive assessment using the Kinarm: an interactive robotic device that uses a series of behavioral tasks involving movement of the upper limbs to precisely quantify neurocognitive impairment across a variety of neurocognitive domains.

**Methods:**

Multilevel modeling was used to determine the effect of Kinarm task type, kidney diagnostic group (AKI vs CKD vs kidney failure), and the interaction between the two, on neurocognitive performance.

**Results:**

A total of 104 participants within 1 year of an AKI event or with CKD category G3-5 were enrolled. We found that across all of the kidney diagnostic groups, participants performed worst on the Kinarm tasks of Reverse Visually Guided Reaching (*b* = 0.64 [95% confidence interval = 0.42, 0.85]), Visually Guided Reaching (*b* = 0.28 [0.07, 0.49]), and Trail Making (*b* = 0.50 [0.28, 0.72]), relative to all other tasks. There were no significant differences in average performance across tasks based on kidney diagnostic group. However, diagnostic group and neurocognitive task type interacted to determine performance, such that patients with AKI performed worse than those with either CKD or kidney failure on the Reverse Visually Guided Reaching task.

**Limitations:**

Kinarm assessment was performed at a single time point, and the sample size itself was small, which may lead to the risk of a false-positive association despite the use of multilevel modeling. Our sample size also did not permit inclusion of the underlying etiology of kidney impairment as a covariate in our analyses, which may have also influenced neurocognitive function.

**Conclusions:**

In this study that utilized the Kinarm to assess neurocognitive function, patients with AKI demonstrated significantly worse neurocognitive functioning than patients with CKD or kidney failure on a task measuring executive function and visuomotor control.

## Abstract (fr)

**Contexte:**

La déficience neurocognitive est fréquemment observée dans le spectre des maladies rénales et elle entraîne des conséquences importantes sur la qualité de vie. Nous avons précédemment démontré que la technologie robotique peut identifier les troubles neurocognitifs qui ne sont pas facilement détectables par les tests traditionnels chez les patients atteints d’insuffisance rénale aiguë (IRA) et d’insuffisance rénale chronique (IRC).

**Objectif:**

La présente étude visait à déterminer si ces déficits quantifiables dans les fonctions neurocognitives diffèrent selon un diagnostic d’IRA, d’IRC ou d’insuffisance rénale terminale (IRT).

**Type d’étude:**

Analyse transversale des participants précédemment inscrits à une étude observationnelle.

**Cadre:**

Les patients avaient été recrutés dans un hôpital universitaire tertiaire, le Kingston Health Sciences Centre, de Kingston (Ontario) au Canada.

**Sujets:**

Des adultes atteints d’IRA, d’IRC ou d’IRT.

**Mesures:**

Chaque participant a subi une évaluation neurocognitive robotique à l’aide du Kinarm: un dispositif robotique interactif qui utilise une série de tâches comportementales impliquant des mouvements des membres supérieurs pour quantifier avec précision les troubles neurocognitifs dans divers domaines neurocognitifs.

**Méthodologie:**

On a utilisé une modélisation à plusieurs niveaux pour déterminer l’effet du type de tâche Kinarm, du groupe de diagnostic rénal (IRA c. IRC c. IRT), et l’interaction entre les deux, sur la performance neurocognitive.

**Résultats:**

L’étude porte sur les 104 patients atteints d’IRC de stade G3-5 ou ayant vécu un épisode d’IRA dans l’année. Nous avons constaté que, dans tous les groupes de diagnostic, les participants ont obtenu les pires résultats pour les tâches Kinarm de l’atteinte guidée visuellement inversée (*b* = 0,64 [intervalle de confiance à 95 %: 0,42-0,85)), de l’atteinte guidée visuellement (*b* = 0,28 [0,07-0,49]) de création de parcours (*b* = 0,50 [0,28-0,72]), par rapport à toutes les autres tâches. Aucune différence significative n’a été observée dans le rendement moyen entre les tâches selon le diagnostic rénal. Cependant, le groupe de diagnostic et le type de tâche neurocognitive ont interagi pour déterminer les performances, de sorte que les patients atteints d’IRA ont obtenu de moins bons résultats que les patients atteints d’IRC ou d’IRT pour la tâche d’atteinte visuelle inversée guidée.

**Limites:**

L’évaluation Kinarm n’a été effectuée qu’une seule fois, sur un échantillon plutôt faible, ce qui pourrait entraîner un risque d’association faussement positive malgré l’utilisation d’une modélisation à plusieurs niveaux. La taille de notre échantillon n’a pas permis d’inclure l’étiologie sous-jacente de l’atteinte rénale comme covariable dans nos analyses, ce qui aurait pu également influencer la fonction neurocognitive.

**Conclusion:**

Dans cette étude où le Kinarm a été utilisé pour évaluer la fonction neurocognitive, les patients atteints d’IRA ont montré des fonctions neurocognitives significativement inférieures à celles des patients atteints d’IRC ou d’IRT lors de tâches mesurant la fonction exécutive et le contrôle visuomoteur.

## What was known before

- Kidney disease is increasingly common and associated with long-term neurocognitive impairment.
- Robotic technology can precisely quantify subtle neurocognitive declines in patients with varying degrees of kidney impairment and has previously allowed for detection of visuomotor and executive function impairment in patients with acute kidney injury (AKI) and chronic kidney disease (CKD).
- No studies to date have explored the differences in cognitive declines between patients with different kidney disease severities.

## What this adds

- This study sought to understand and quantify the differences in neurocognitive function between patients with diagnosed AKI, CKD, and kidney failure using robotic technology.
- We found that, although patients with AKI, CKD, and kidney failure exhibit similar degrees of global neurocognitive impairment, patients with a recent history of an AKI event had even more impairment on tasks of visuomotor and executive function using the Kinarm, compared to individuals with CKD/kidney failure.

## Introduction

Kidney disease, including acute dysfunction (acute kidney injury, AKI) and long-term sustained impairment (chronic kidney disease, CKD) or kidney failure, is common among Canadians.^1,2^ Almost one-third of adults admitted to Canadian intensive care units (ICUs) ultimately develop AKI,^1^ whereas 12.5% of Canadians are currently living with CKD,^2^ and nearly 41 000 Canadians have kidney failure.^3^ Kidney disease is associated with a wide array of comorbidities affecting quality of life.^4^ Kidney disease is known to be detrimental to neurocognitive functioning,^5,6^ likely as a consequence of increased accumulation of uremic toxins, vascular injury, and endothelial dysfunction.^7^ Early detection of neurocognitive impairments is critical to being able to offer supports to this vulnerable patient population.

Although traditional assessments of neurocognitive function have relied on a variety of validated test batteries and screening tools,^8^ robotic technology is more sensitive in detecting subtle neurocognitive impairment.^9^ In patients with CKD and AKI, robotic technology has been able to quantify both profound and more subtle neurocognitive impairments.^10,11^ This impairment was specifically seen in complex tasks of perceptual motor skills, executive function, and attention.^10^ Impairments in these areas are corroborated by more recent literature supporting the decline of visuomotor and executive function early on in patients with kidney disease.^12,13^

Although neurocognitive impairment in CKD and kidney failure is well established, studies examining and comparing objective and quantifiable neurocognitive impairments across the full spectrum of kidney disease are lacking. Furthermore, given that AKI has also been recently associated with neurocognitive impairment, it is important to contextualize the degree of this impairment with the deficits observed in patients with CKD and kidney failure, as these clinical populations are commonly seen and assessed in nephrology clinics. A heightened awareness for the potential of neurocognitive dysfunction in these individuals is important, as it raises critical questions regarding driving safety, medication adherence, and medical decision making.

The Kinarm end-point (EP) (Kinarm, Kingston, Ontario, Canada) is a robotic technology designed to specifically and precisely quantify neurocognitive impairment across a variety of cognitive domains, using Kinarm Standard Tests™ (KST). This study aimed to assess variation in neurocognitive impairment as a function of Kinarm task type and kidney diagnostic group. Specifically, we sought to understand whether patients with AKI, CKD, and kidney failure demonstrated comparable differences in neurocognitive performance depending on task type, and whether this pattern differed between patients with AKI vs CKD vs kidney failure. We hypothesized that participants with any kidney dysfunction would perform more poorly on tasks involving executive function and control (ie, Kinarm tasks: Reverse Visually Guided Reaching [RVGR] and Trail Making [TM]) than on tasks that do not involve these higher order neurocognitive functions. Furthermore, we hypothesized that this effect would be moderated by disease severity, with participants with more severe kidney dysfunction demonstrating a higher degree of neurocognitive impairment.

## Materials and Methods

### Study Design

This was a single-center, retrospective, secondary cross-sectional analysis of participants with diagnosed kidney disease who were enrolled into a prospective observational cohort study for a different purpose at Kingston Health Sciences Centre (KHSC; Kingston, Ontario, Canada). A subset of participants enrolled into the prospective observational cohort study with AKI and CKD have been previously reported.^10,11^ The present study expands upon these participant cohorts and combines data from all Kinarm tasks and kidney diagnostic categories to compare performance of patients across a range of neurocognitive tasks.

### Participants

A convenience sample of participants were eligible for inclusion into the prospective observational cohort study if they were greater than 17 years of age and had AKI, CKD, or kidney failure. Acute kidney injury was defined using the Kidney Disease Improving Global Outcomes (KDIGO) serum creatinine criteria,^14^ and participants in this group were enrolled within 1 year of their AKI event. Chronic kidney disease was defined as category G1-5 based on estimated glomerular filtration rate (eGFR), in accordance with KDIGO CKD guidelines.^15^ Kidney failure was defined as an eGFR <15 mL/min/1.73 m^2^. Participants were excluded if they had a documented history of stroke, neurodegenerative disease, psychiatric illness, or uncorrected vision loss, which could affect their performance on tests of neurocognitive functioning independent of their kidney disease.

### Ethics Approval

This study was reviewed and approved by the Queen’s University and Affiliated Teaching Hospitals Health Sciences Research Ethics Board (Approval #DMED-1784-15) and was performed in accordance with the ethical standards laid down in the Declaration of Helsinki (as revised in Brazil 2013). All participants provided written informed consent prior to participating in this study.

### Neurocognitive Assessment

All consented participants were assessed for neurocognitive impairment either at the time of enrollment or during one of their subsequent ambulatory clinical follow-up visits. Participants completed a series of 8 neurocognitive tasks on the Kinarm EP robot, as outlined in Supplementary Table 1. The order in which these tasks were performed was consistent across all participants. These KSTs automatically quantify parameter scores, which define the spatial and temporal characteristics of performance for each of the 8 tasks and are adjusted for age, sex, and handedness.^16^ These parameters are then used to generate standardized task scores for each task, with higher scores indicating poorer performance on the task. Task scores of 0 indicate the best possible performance on the task, whereas task scores of 1 and 1.96 represent the 68th and 95th percentile, respectively, based on a large cohort of healthy control participants. Task scores greater than 1.96 are considered to be outside the normal range of performance.

### Data Collection

Demographics (age and sex) and data on participants’ kidney function (serum creatinine), need for dialysis, dialysis modality and vintage, number of hospital and ICU admissions, and past medical history of hypertension and diabetes (Type 1 and Type 2) were collected from the electronic medical record. Serum creatinine values were obtained from the clinical visit closest in time to the participant’s neurocognitive assessment. Ethnicity was obtained by participant self-report during the study visit.

### Statistical Analysis

All analyses were performed using the R statistical software package.^17^ Descriptive statistics were calculated using mean (standard deviation, SD) for continuous variables or number (percentage) for categorical variables. Estimated glomerular filtration rate was used to classify each participant into the CKD and kidney failure diagnostic categories, in accordance with KDIGO guidelines for the classification of CKD/kidney failure.^15^ Participants who met the criteria for AKI based on KDIGO guidelines^14^ were classified into the AKI group. Participants’ baseline Kinarm task scores were modeled as a function of task type and diagnostic group (AKI, CKD [category G1-4], or kidney failure [CKD category G5]). We conducted a multilevel modeling (MLM) analysis using the *nlme* package in R^18^ to compare the burden of neurocognitive impairment on different neurocognitive tasks across patients in the different kidney diagnostic groups, treating the neurocognitive assessment measures (Kinarm task scores) as the outcome of interest. The methodology describing the rationale for model selection for statistical analysis can be found in the Supplemental Material.

### Availability of Data and Materials

The data underlying this article will be shared on reasonable request to the corresponding author.

## Results

### Participant Characteristics and Data Collection

**Figure 1.**
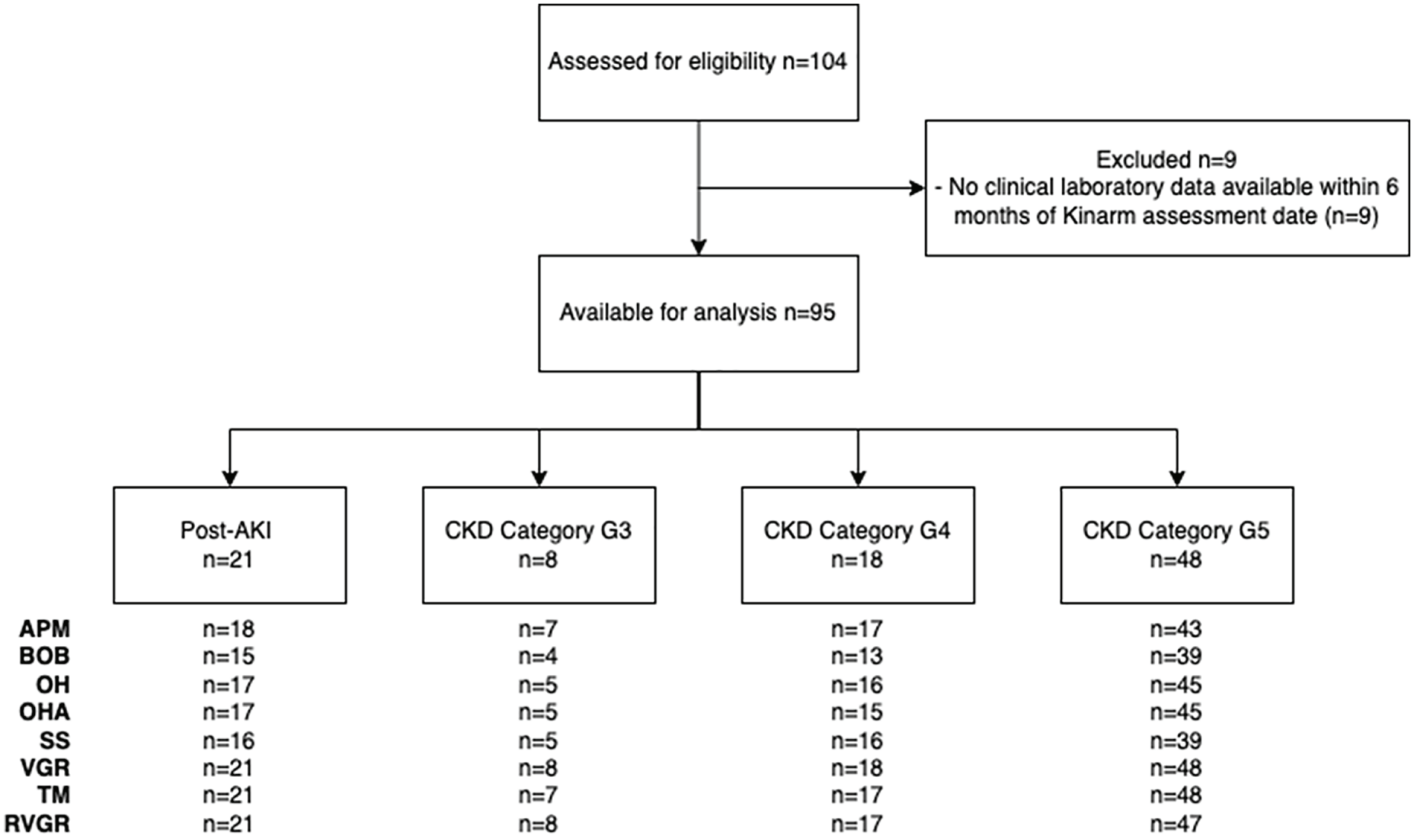
Participant flow diagram. *Note.* Missing Kinarm data was primarily due to patients having time constraints and not being able to stay for the whole assessment. AKI = acute kidney injury; CKD = chronic kidney disease; APM = arm position matching; BOB = ball on bar; OH = object hit; OHA = object hit and avoid; SS = spatial span; VGR = visually guided reaching; TM = trail making; RVGR = reverse visually guided reaching.

A total of 104 participants were enrolled into this study. Nine participants were excluded from the analysis as they had no available clinical laboratory data within 6 months of their baseline Kinarm assessment date and therefore no eGFR could be calculated. Data were available on 21 participants in the AKI group, 26 in the CKD group, and 48 in the kidney failure group. Participant data and exclusions are summarized in the study flowchart (Figure 1). All but 5 participants identified as White (94.7%), and 69.5% were male. Baseline demographic and clinical data are outlined in Table 1.

**Table 1.** Data at the Time of Cognitive Assessment.

	Post-AKI	Baseline CKD category		
		G3	G4	G5 (kidney failure)
Number of participants	21	8	18	48
Age (years)	70.95 (8.02)	68.50 (14.08)	67.94 (16.56)	62.98 (12.79)
Male sex	16 (76.19)	7 (87.50)	13 (72.22)	30 (62.50)
eGFR (mL/min/1.73 m^2^)	37.76 (21.56)	37.12 (8.29)	21.44 (4.13)	10.52 (3.53)
Serum creatinine (μmol/L)	191.87 (121.94)	160.38 (24.70)	246.44 (54.42)	475.75 (177.08)
Time from diagnosis (months)	6.29 (4.28)	7.25 (5.42)	35.33 (30.81)	46.06 (43.96)
Dialysis	12	0	1	12
iHD	9	0	1	9
PD	0	0	0	3
iHD + PD	0	0	0	0
CKRT and iHD	3	0	0	0
Dialysis vintage (days)	231.58 (142.59)	0 (0.00)	307.83 (416.97)	588.98 (533.09)
Hypertension	2	2	12	35
Diabetes	4	2	7	33
Type 1	0	0	0	5
Type 2	4	2	7	28
Hospitalizations	13	1	5	10

*Note.* Mean (SD) or n (%). Dialysis for the post-AKI group is historic from the time of the AKI event; all other data is from the time of cognitive assessment. AKI = acute kidney injury; CKD = chronic kidney disease; eGFR = estimated glomerular filtration rate; iHD = intermittent hemodialysis; PD = peritoneal dialysis; CKRT = continuous kidney replacement therapy.

Two-thirds (14/21) of participants with AKI had been admitted to an ICU during their AKI event, and 11/21 were initiated on kidney replacement therapy during their inpatient hospital admission as a result of their AKI (8 on intermittent hemodialysis [iHD], 2 on continuous kidney replacement therapy [CKRT], and one who was started on CKRT and later transitioned to iHD).

### The Proportion of Participants Categorized as Impaired Varies Depending on the Kinarm Task

**Figure 2.**
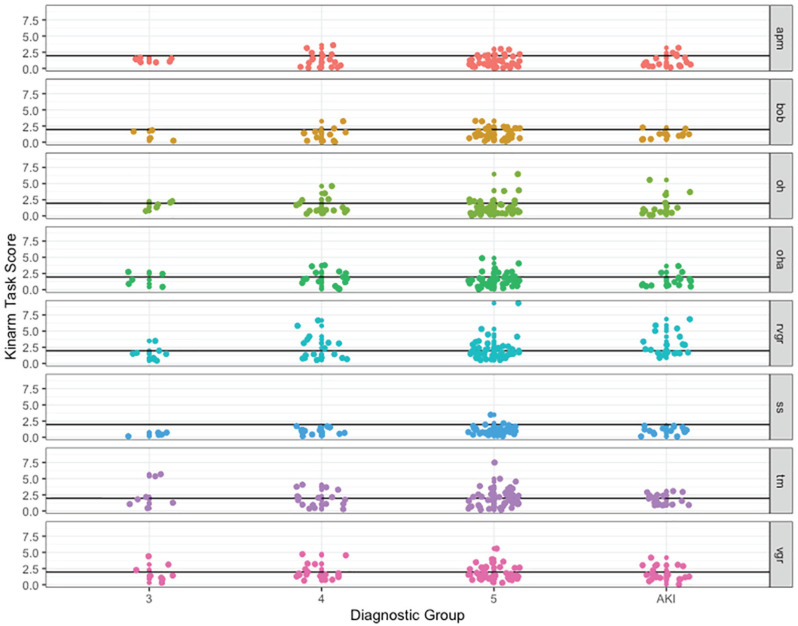
Kinarm task scores by diagnostic group. *Note.* Black line indicates the 95th percentile score for healthy controls on each task; scores above the black line are considered to be outside of the normal range. APM = arm position matching; BOB = ball on bar; OH = object hit; OHA = object hit and avoid; RVGR = reverse visually guided reaching; SS = spatial span; TM = trail making; VGR = visually guided reaching; AKI = acute kidney injury.

Kinarm task scores by kidney disease diagnostic group and task are depicted in Figure 2. Overall numbers of participants in each diagnostic group who scored outside the 95th percentile are presented in Table 2. Across all diagnostic groups, the highest degree of impairment was on the TM and the RVGR tasks. Specifically, 42 (45.2%) participants were impaired on the TM task; 38 (40.9%) on RVGR; 31 (32.6%) on VGR; 23 (28.0%) on object hit and avoid (OHA); 19 (22.9%) on object hit (OH); 14 (19.7%) on ball on bar (BOB); 14 (16.5%) on arm position matching (APM); and 2 (2.6%) on spatial span (SS).

**Table 2.** Neurocognitive Impairment on Kinarm Tasks.

Task	Post-AKI		CKD G3		CKD G4		CKD G5 (kidney failure)	
	N impaired (%)	N total	N impaired (%)	N total	N impaired (%)	N total	N impaired (%)	N total
Arm position matching	3 (16.7)	18	0 (0)	7	4 (23.5)	17	7 (16.3)	43
Ball on bar	2 (13.3)	15	0 (0)	4	2 (15.4)	13	10 (25.6)	39
Object hit	4 (23.5)	17	2 (40.0)	5	4 (25.0)	16	9 (20.0)	45
Object hit and avoid	3 (17.6)	17	2 (40.0)	5	6 (40.0)	15	12 (26.7)	45
Spatial span	0 (0)	16	0 (0)	5	0 (0)	16	2 (5.1)	39
Visually guided reaching	7 (33.3)	21	3 (37.5)	8	6 (33.3)	18	15 (31.3)	48
Trail making	9 (42.9)	21	3 (42.9)	7	8 (47.1)	17	22 (45.8)	48
Reverse visually guided reaching	11 (52.4)	21	2 (25.0)	8	8 (47.1)	17	17 (36.2)	47

*Note.* AKI = acute kidney injury; CKD = chronic kidney disease; N = number of participants.

#### Patients with AKI, CKD, and kidney failure perform poorly on tasks of visuomotor and executive function

To assess the association between Kinarm task performance and category of kidney disease, the random intercepts model was used (see Supplementary Results). At the average of the diagnostic groups, we determined the effect of task type on neurocognitive performance. Results are shown in Table 3. Compared to their mean performance across all Kinarm tasks (the grand mean), participants performed significantly worse on the RVGR task, *b* = 0.65 [95% confidence interval = 0.43, 0.87], on the VGR task, *b* = 0.29 [0.08, 0.51], and on the TM task, *b* = 0.49 [0.27, 0.72]. Participants performed significantly better relative to the grand mean for all Kinarm tasks on the BOB task, *b* = −0.36 [−0.63, −0.08], and on the APM task, *b =* −0.33 [−0.56, −0.10]. No significant differences relative to the grand mean of all tasks were found on the OH task or the OHA task.

**Table 3.** Effect of Task Type on Neurocognitive Performance Across All Participants.

Task	*b*	SE	*t*	*p*	CI
RVGR	0.65	0.11	5.73	<.001	0.43 to 0.87
VGR	0.29	0.11	2.59	.01	0.07 to 0.51
TM	0.49	0.12	4.22	<.001	0.27 to 0.72
BOB	−0.36	0.14	−2.49	.01	−0.63 to −0.08
APM	−0.33	0.12	−2.79	.005	−0.56 to 0.10
OH	−0.12	0.13	−0.91	.36	−0.37 to 0.13
OHA	−0.02	0.13	−0.16	.87	−0.27 to 0.23

*Note. b* = beta coefficient; *t* = *t* statistic; *p* = *p*-value; CI = confidence interval; RVGR = reverse visually guided reaching; VGR = visually guided reaching; TM = trail making; BOB = ball on bar; APM = arm position matching; OH = object hit; OHA = object hit and avoid.

#### Kidney diagnostic group does not affect global neurocognitive function

At the average of the Kinarm tasks, none of the means for the kidney diagnostic group were significantly different from the grand mean of all diagnostic groups: AKI group, *b* = −0.02 [−0.30, 0.26], CKD category G4 group, *b* = 0.10 [−0.20, 0.39], CKD category G5 group (kidney failure), *b* = 0.01 [−0.23, 0.22].

#### Kidney diagnostic group moderates performance on a task of visuomotor and executive function

**Figure 3.**
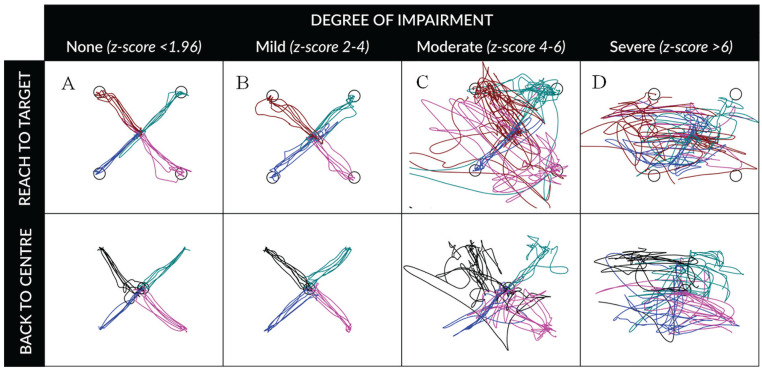
Hand path tracings for varying degrees of impairment on the Reverse Visually Guided Reaching task. *Note.* The Reverse Visually Guided Reaching task assesses attention, inhibitory control, and cognitive control of visuomotor skills. The participant controls a cursor on screen and is asked to first reach out toward a target and then return back to the center. The movement of the cursor is reversed compared to their actual hand position by 180 degrees. The participant must therefore override their normal response to move their hand directly to the target, and instead, initiate a movement in the exact opposite direction. Figure 3 depicts the hand path tracings of participants with kidney disease completing the task, at varying degrees of impairment. Panel A depicts an individual with no demonstrable impairment; panel B mild impairment; panel C moderate impairment; and panel D severe impairment.

A significant interaction between task and kidney disease severity was found for the RVGR task only, in that participants with AKI had significantly poorer performance scores relative to the grand mean of all diagnostic groups, *b* = 0.63 [0.28, 0.97]. None of the other interactions were significant. The simple effect of being in the AKI group and the simple effect of being in any other diagnostic group for the RVGR task were then examined to follow-up on the significant interaction.^19^ Participants in both the AKI and the non-AKI groups performed worse on the RVGR task relative to the grand mean for all Kinarm tasks; however, participants in the AKI group performed more poorly than did those without AKI, (*b* = 0.94 [0.62, 1.25] vs *b* = 0.51 [0.34, 0.67] for the AKI and no-AKI groups, respectively). Examples of hand path tracings while performing RVGR are shown in Figure 3. An individual with no demonstrable impairment (*z*-score <1.96, Figure 3A) reaches out to the target and back directly in a relatively straight line, with little or no overshoot or direction error. An individual with a mild degree of impairment (eg, *z*-score 2-4, Figure 3B) has increased variability in trajectory to the target from trial to trial, but still is able to get the cursor to the target and back. As the degree of impairment worsens to moderate (*z*-score 4-6, Figure 3C) and severe (*z*-score >6), the hand path trajectories become increasingly random and inconsistent, with clear failure to reach the target. Some of the most impaired individuals appear to move the cursor in a random pattern (Figure 3D).

## Discussion

Visuospatial and executive dysfunction are being increasingly reported in patients with kidney disease.^10,12,13^ The aim of this study was to extend our preliminary findings suggesting that robotic technology can quantify subtle neurocognitive impairments in patients with CKD^10^ and after a single episode of AKI.^11^ Specifically, we wanted to assess the association between degree of kidney dysfunction and quantitative metrics of neurocognitive performance. This single-center study analyzed data from patients previously enrolled in a prospective observational study, where neurocognitive function was assessed using robotic technology.

We found a significant effect of task type on neurocognitive functioning, such that participants performed worst on the RVGR, VGR, and TM tasks relative to their performance on the other tasks. This finding is consistent with previous literature suggesting that perceptual motor/visuomotor functioning, executive functioning, and attention are impaired in patients with kidney dysfunction.^10^

In addition, we found a significant interaction between task type on the RVGR task and kidney diagnostic group (AKI vs grand mean of all diagnostic groups) on neurocognitive performance. This may indicate that kidney diagnostic group modifies the relationship between task type and neurocognitive functioning when examining participants with AKI compared to participants with CKD/kidney failure. Although all participants performed poorly on the task, those with AKI performed even worse, in contrast to our a priori hypothesis. This suggests that recent history of an AKI event within the past 1 year may lead to even more impairment on tasks of visuomotor and executive function using the Kinarm, compared to individuals with CKD/kidney failure. However, this may be related to other aspects of the patients’ condition beyond their kidney function. Importantly, two-thirds (14/21) of participants with AKI had their AKI event in the context of a critical illness requiring ICU admission, and more than half (11/21) received kidney replacement therapy while hospitalized. Critical illness and treatment with dialysis are each independently associated with lasting neurocognitive impairment.^20,21^ The combined effects of kidney disease, critical illness, and dialysis among the majority of AKI participants enrolled in this study may explain the increased degree of impairment experienced by patients following their AKI event.

A recent review found that patients with CKD are at a significant risk for unsafe driving as a result of their impaired cognition, and up to one-third of patients on hemodialysis were involved in motor vehicle collisions since initiation of dialysis.^22^ Driving is just one of the many implications of cognitive dysfunction in this patient population—these patients are also often on multiple medications and required to manage their diet and fluid intake, all of which encompass their instrumental activities of daily living (IADLs). The profound extent of impairment in neurocognitive function is exemplified by the hand-path tracings of participants performing the RVGR task in our study (Figure 3).

Visuomotor skills, executive function, and attention are all critical for performing both basic activities of daily living (ADLs) and IADLs.^23-25^ Activities of daily living are fundamental skills that are required to care for oneself (eg, bathing, dressing, toileting, transfer, continence, and feeding).^26,27^ Instrumental activities of daily living, on the contrary, are more complex adaptive skills that enable one to live independently (eg, shopping, cooking, housekeeping, managing finances, managing medications).^27^ The ability to perform IADLs is associated with greater quality of life, despite not being required for daily functioning.^27,28^ In patients with kidney disease, both ADLs and IADLs are known to be compromised.^29^ The neurocognitive deficits we found in our study may therefore contribute to the decline in overall quality of life in patients with kidney disease.

Our study’s limitations include the cross-sectional nature of the data, and the small sample size of participants in each diagnostic group. The use of MLM allowed us to preserve a higher power in our statistical analyses, despite our sample size limitations; however, there is still an inherent risk of type I and type II error with the limited sample size. Multilevel modeling was selected due to the hierarchical structure of the data, in that Kinarm tasks were nested within patients. That is, within each patient, the neurocognitive test measurements in each task may be contingent on the performance in other tasks, resulting in correlation among observations within each participant. Averaging across the groups to look at differences between tasks only, or averaging between tasks to look at differences between groups only, would ignore the multilevel hierarchical structure of the data, inflating the Type I error rate and reducing statistical power.^30^ Multilevel modeling allowed us to examine performance on the various tasks through a single analysis, while taking into account the dependence in task performance. Moreover, MLM has been shown to outperform other within-subjects analyses like repeated-measures analysis of variance (ANOVA) and multivariate analysis of variance (MANOVA) in terms of Type I error and power and has other advantages including allowing for missing data and less stringent assumptions (eg, sphericity).^31^

The inability to match the underlying characteristics of each of the kidney diagnostic categories is a limitation of our study. Our study also made use of the epidemiology collaboration (CKD-EPI) equation for eGFR, which accounts for participants’ race. There is currently a move away from including race in eGFR equations; however, since the majority of our cohort was white, race likely did not play an important factor in determining eGFR in our study. Our sample size also did not permit inclusion of the underlying etiology of kidney impairment as a covariate in our analyses, which may have also influenced patient’s neurocognitive function.

## Conclusions

Overall, our study demonstrates the importance of evaluating patients for neurocognitive impairment across the spectrum of kidney disease, as well as patients after a single AKI event. Particular attention should be taken in assessing the neurocognitive domains involving perceptual motor skills, executive functioning, and attention in these cohorts. As AKI follow-up clinics are becoming more common in centers across North America, our study suggests that screening for cognitive impairment in this vulnerable patient population would be an important component of their clinical assessment.

## Supplemental Material

sj-docx-1-cjk-10.1177_20543581221103100 – Supplemental material for Visuospatial and Executive Dysfunction in Patients With Acute Kidney Injury, Chronic Kidney Disease, and Kidney Failure: A Multilevel Modeling Analysis Click here for additional data file. Supplemental material, sj-docx-1-cjk-10.1177_20543581221103100 for Visuospatial and Executive Dysfunction in Patients With Acute Kidney Injury, Chronic Kidney Disease, and Kidney Failure: A Multilevel Modeling Analysis by Natasha A. Jawa, Jessica A. Vanderlinden, Stephen H. Scott, Jill A. Jacobson, Samuel A. Silver, Rachel Holden and J. Gordon Boyd in Canadian Journal of Kidney Health and Disease
