# Supplementary material for: Visuospatial and Executive Dysfunction in Patients With Acute Kidney Injury, Chronic Kidney Disease, and Kidney Failure: A Multilevel Modeling Analysis
Source: Can J Kidney Health Dis. 2022 Jun 14;9:20543581221103100. doi: 10.1177/20543581221103100 (PMC9201347; doi:10.1177/20543581221103100)
Supplement: sj-docx-1-cjk-10.1177_20543581221103100 – Supplemental material for Visuospatial and Executive Dysfunction in Patients With Acute Kidney Injury, Chronic Kidney Disease, and Kidney Failure: A Multilevel Modeling Analysis [file sj-docx-1-cjk-10.1177_20543581221103100.docx]

**Supplementary Methods:**

Coding and data analysis:

For this MLM analysis, the Level 1 predictor variable representing the data within each participant was the Kinarm task type. The Level 2 predictor variable representing data between participants was the kidney diagnostic group (AKI *vs.* CKD *vs.* ESKD). Prior to the analysis, all categorical predictor variables were coded.

While multiple coding systems exist, we opted to use effects coding for our categorical predictor variables, as this was an observational study with no control group (despite each participants’ task scores being normalized to a healthy control Kinarm dataset). Kinarm tasks were effects coded according to *Supplementary Table 2*, such that participants’ performance on each task (the variable coded as 1) was compared to the grand mean of participants’ performance across all tasks. For example, for the first task analysis in *Supplementary Table 2*, the RVGR task is coded as 1, and therefore this analysis represents the effect of the RVGR task. Diagnostic groups were effects coded according to *Supplementary Table 3*.

When using an effects coding system, one of the levels of the predictor variable (i.e., diagnostic group or task type) must be dropped from the analysis, by coding that level as -1. For task type, the level that was dropped was the spatial span (SS) task, as it was previously found to be less sensitive at detecting impairment in patients with kidney disease (10). For diagnostic group, the level that was dropped was the CKD category 3 group, as our goal was to compare later-category CKD, ESKD, and AKI.

For this MLM analysis an unstructured covariance matrix was used along with the between-within method of estimating degrees of freedom, and the maximum likelihood method for estimating the model. The maximum likelihood method was used to allow for comparison of the two models using the chi-square difference test.

Model selection:

We used two models to examine the variation in neurocognitive impairment (as determined by Kinarm performance) by kidney diagnostic group. The first model allowed patients’ mean performance on the Kinarm tasks (i.e., intercepts) to vary for each of the diagnostic groups, while keeping the relationship (i.e., slopes) between the diagnostic group and performance consistent across patients (random intercepts model). The second model allowed both the intercepts and slopes to vary for each diagnostic group (random intercepts and slopes model).

The random intercept and random intercept and slope models were compared using a chi-square difference test. Reported values are from the preferred model. Significant interaction effects were followed up by examining the simple effect of diagnostic group for the relevant task.

**Supplementary Results:**

The results of the two-level multilevel modeling analysis using the random intercepts model showed significant heterogeneity in patients’ mean Kinarm task scores, 95% confidence interval for the intercept [0.86, 0.97], which supports allowing the mean performance on the Kinarm tasks (i.e., intercepts) to vary for each of the diagnostic groups and lends credence to our use of a random intercepts model for this analysis.

The results of the random intercepts and slopes model showed that there was significant heterogeneity of variance in patients’ mean Kinarm task scores (i.e., intercepts) and in the relationship between neurocognitive performance and diagnostic group (i.e., slopes; 95% confidence intervals did not contain zero). However, the covariance between the means and the slopes was not significant (95% confidence interval did contain zero, [-1.00, 1.00]); therefore there was no consistent pattern by which performance on the Kinarm tasks changed across the different diagnostic groups.

The random intercepts model was preferred over the random intercepts and slopes model, as there was no significant difference between the models on the chi-square difference test, *χ*^2^ = .19, *p =*.91, and the random intercepts model was more parsimonious, fitting the data equally well by using fewer predictor variables.

**Supplementary Table 1.** Kinarm tasks and associated neurocognitive domains.

| Task | DSM-5 neurocognitive domain(s) | Purpose of test |
| --- | --- | --- |
| Arm position matching | Perceptual motor | Provides a measure of the participant’s proprioceptive/position-sense capabilities. The robot moves one of the participant’s arms to a given position, and the participant must mirror-match the position with their other arm. |
| Ball on bar | Perceptual motor | Assesses bimanual coordination by having the participant balance a ball on a bar, with progressive levels of difficulty. |
| Object hit | Perceptual motor, complex attention | Tests sensorimotor, decision, and control of the participant by using paddles to push away balls at progressively faster speeds. |
| Object hit and avoid | Percentual motor, complex attention | Requires executive function emphasizing attention, rapid motor selection, and inhibition by having the participant use paddles to push away only two target shapes and ignore all distracter shapes. |
| Spatial span | Learning and memory | Evaluates visuospatial working memory by illuminating squares in a 3x4 grid in a particular sequence and having the participant repeat back the sequence, in progressively longer trials. |
| Visually guided reaching | Perceptual motor | Assesses visuomotor capabilities by having the participant move the cursor to a control target quickly and accurately. |
| Reverse visually guided reaching | Perceptual motor, complex attention, executive function | Assesses cognitive inhibition by having the participant move the cursor to the mirror-matched position from the target. |
| Trail making test A/B | Perceptual motor, complex attention, executive function | Assesses attention, visuomotor function, and processing speed by having the participant complete a series of traces through numeric targets (1-25; Trail A), and alternative alpha-numeric targets (1-A-2-B, etc. up to 25 targets; Trail B). |

Adapted with permission from the Kinarm User Guide[21] and Vanderlinden et al.[14]. DSM-5, diagnostic and statistical manual of mental disorders, 5^th^ edition.

**Supplementary Table 2.** Effects coding for Kinarm tasks.

| Task | E1 | E2 | E3 | E4 | E5 | E6 | E7 |
| --- | --- | --- | --- | --- | --- | --- | --- |
| RVGR | 1 | 0 | 0 | 0 | 0 | 0 | 0 |
| VGR | 0 | 1 | 0 | 0 | 0 | 0 | 0 |
| OH | 0 | 0 | 1 | 0 | 0 | 0 | 0 |
| OHA | 0 | 0 | 0 | 1 | 0 | 0 | 0 |
| TM | 0 | 0 | 0 | 0 | 1 | 0 | 0 |
| BOB | 0 | 0 | 0 | 0 | 0 | 1 | 0 |
| APM | 0 | 0 | 0 | 0 | 0 | 0 | 1 |
| SS | -1 | -1 | -1 | -1 | -1 | -1 | -1 |

E1, effects coded contrast 1; E2, effects coded contrast 2; E3, effects coded contrast 3; E4, effects coded contrast 4; E5, effects coded contrast 5; E6, effects coded contrast 6; E7, effects coded contrast 7; RVGR, reverse visually-guided reaching; VGR, visually-guided reaching; OH, object hit; OHA, object hit and avoid; TM, trail making; BOB, ball on bar; APM, arm position matching; SS, spatial span.

**Supplementary Table 3.** Effects coding for diagnostic groups.

| Diagnostic group | E1 | E2 | E3 |
| --- | --- | --- | --- |
| Post-AKI | 1 | 0 | 0 |
| CKD G3 | -1 | -1 | -1 |
| CKD G4 | 0 | 1 | 0 |
| CKD G5/kidney failure | 0 | 0 | 1 |

E1, effects coded contrast 1; E2, effects coded contrast 2; E3, effects coded contrast 3; AKI, acute kidney injury; CKD, chronic kidney disease.
